# Optimization of ultrasound‐assisted extraction method for phytochemical compounds and antioxidant activities of sour jujube extracts

**DOI:** 10.1002/fsn3.2971

**Published:** 2022-08-03

**Authors:** Yanyan Wang, Wan Zhao, Yixiang Li, Hang Zhao, Xiaonan Ye, Tingli Li, Zhibin Wang, Lili Huang

**Affiliations:** ^1^ Department of Pharmacology of Traditional Chinese Medicine Heilongjiang University of Chinese Medicine Harbin China; ^2^ Department of Pharmacy Heilongjiang Provincial Hospital Harbin China

**Keywords:** antioxidants, central composite design, sour jujube, ultrasonic‐assisted extraction

## Abstract

Ultrasonic‐assisted extraction is a rapid and effective extraction method that uses ultrasound energy and solvents to extract target compounds from various plant matrices. In this study, the ultrasonic‐assisted extraction conditions of sour jujube were optimized. A five‐level central composite design (CCD) with four variables was used to evaluate ultrasonic treatment variables influencing the total saponin content (TSC), total flavonoid content (TFC), and total phenolic content (TPC) extracted from sour jujube. The solvent concentration, extraction time, ultrasonic power, and solid‐to‐liquid (S/L) ratio were optimized using aqueous ethanol and methanol solutions as extraction solvents. A central composite design (CCD) was used for an in‐depth study, and then the optimal value that could produce the maximum TPC, TFC, TSC, and four in vitro antioxidant activities (scavenging activity of hydroxyl free radicals, ferric‐reducing antioxidant power (FRAP), phosphomolybdic acid reduction method, and 1,1‐diphenyl‐2‐picrylhydrazyl (DPPH) radical‐scavenging activity) was determined. Hydrogen peroxide‐induced oxidative stress experiment confirmed that the Jujube extract could have an antioxidant role in vivo. The relationship between the contents of three compounds and the antioxidant activity in vitro and in vivo was further studied. The results showed that optimizing methanol and ethanol extraction process parameters could improve target components’ extraction efficiency. Under the optimum conditions, the TFC and TPC yields of sour jujube by ethanol are better than methanol, while the yield of TSC by methanol is better than ethanol. In vivo data showed that Jujube extract protects against the adverse effects of oxidative stress and improves the life span of female and male *Drosophila*. This study provides a valuable reference for the full use of *Ziziphus jujube*, as well as a new direction in food development.

## Graphical abstract

1.Sour jujube could be explored as a new dietary supplement to be a potential antioxidant. 2.sour jujube extracts exter excellent antioxidant in vivo and in vitro tests. 3.Determines the preparation process of the antioxidant effective parts of sour jujube and the antioxidant activity of different parts of different origins.

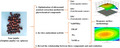

## 1 INTRODUCTION

Sour jujube (also known as Suanzao) is a tree species of *Ziziphus jujuba Mill. var. spinosa (Bunge) Hu ex H.F.Chou* with a high tolerance to cold, drought, waterlogging, and barren. It is widely distributed in Shanxi, Gansu, Liaoning, and other provinces of China. Besides being used as food, the fruit has also been used as medicine for more than two thousand years. Semen *ziziphi spinosae*, a seed of sour jujube, is an important traditional Chinese medicine (Erenmemisoglu et al., 2011; Li et al., 2006). Sour jujube contains a variety of bioactive substances, such as sour jujube polysaccharides, phenols, flavonoids, saponins, triterpenoids, and alkaloids, which are used as folk medicines. As a functional food, its pharmacological activity has attracted increasing attention (Song et al., 2020; Sun et al., 2013).

Free radicals are a major factor associated with a variety of chronic degenerative diseases, tumor, cardiovascular disease, and age‐related functional decline, such as Parkinson’s and Alzheimer's diseases (Jiang et al., 2016). Han et al. found that the Jujube extract has antioxidant activity and can scavenge 1,1‐diphenyl‐2‐picrylhydrazyl (DPPH) and superoxide radicals (Han et al., 2015). DPPH and 2,2′‐Azino‐bis (3‐ethylbenzthiazoline‐6‐sulfonic acid) (ABTS) radical‐scavenging capacity and ferric‐reducing antioxidant power (FRAP) are significantly related to total phenolic contents (TPCs) (Choi et al., 2011; Xue et al., 2010; Zhang et al., 2010). Furthermore, Zhang et al. discovered that triterpenoids and flavonoids isolated from sour jujube have strong antioxidant activity, showing effective free radical‐scavenging activity (Zhang et al., 2014). However, the relationship between different kinds of chemical components and the antioxidant effect of sour jujube is yet to be confirmed.

Ultrasound‐assisted extraction is based on the cavitation process of sound waves that produce a mechanical effect, rupturing cavitation bubbles near or on the surface of plant cell walls, thereby increasing the contact surface area between solids and liquids, which in turn promotes the diffusion of cellular material and solvent to cells (Palsikowski et al., 2020). Its major advantages are high extraction rate, use of low temperature, and shorter processing time, as well as reduced extraction time and solvent consumption (Chemat et al., 2017; Periche et al., 2015; Wen et al., 2018; Yilmaz et al., 2020). Nonetheless, the optimization of the ultrasonic extraction process of sour jujube has not appeared in domestic and foreign literature. In this study, the extraction conditions of sour jujube were optimized. A five‐level central composite design (CCD) with four variables was used to evaluate ultrasonic treatment variables influencing the total saponin content (TSC), total flavonoid content (TFC), and total phenolic content (TPC) extracted from sour jujube. In vitro antioxidant capacity was investigated through hydroxyl radical (•OH) scavenging, total reduction ability, DPPH scavenging, and total antioxidant ability. The effect of Jujube extract on the life span of *Drosophila* under H_2_O_2_ oxidative stress was evaluated.

## 2 MATERIALS AND METHODS

### 2.1 Chemicals and instruments

Salicylic acid, ferric chloride, ammonium molybdate, trisodium phosphate, ferrous sulfate, potassium ferricyanide, and trichloroacetic acid were from Tianjin Yongsheng Fine Chemical Co., Ltd.; glacial acetic acid, perchloric acid, vanillin, aluminum nitrate, sodium nitrite, and sodium hydroxide were obtained from Tianjin Kaitong Chemical Reagent Co., Ltd; acetonitrile was from Beijing Dicoma Technology Co., Ltd. (r141442). An analytical high‐performance liquid chromatograph (Waters 2695‐2998), ultrasonic extraction equipment (Jining Tianhua Ultrasonic Electronic Instrument Co., Ltd, THC‐20B), an analytical balance (Mettler Toledo International Trading Co., Ltd., MS105), an electrothermal constant temperature blast drying oven (DHG‐9055 Shanghai Daohan Industrial Co., Ltd.), and a multifunctional microplate reader (BioTek, Synergy 2) were utilized.

### 2.2 Materials

Sour jujube fruits were obtained from a farm in Yangquan city, Shanxi Province, China, in September 2020. Other sour jujube fruits producing areas were Hotan (Xinjiang), Lvliang (Shanxi), Dunhuang (Gansu), Linyi (Shandong), and Chaoyang (Liaoning). The *Drosophila* specimens, a wild‐type Canton S *Drosophila melanogaster*, were provided by the Core Facility of Drosophila Resource and Technology, CEMCS, CAS.

### 2.3 Extraction procedure

Sour jujube samples were prepared as follows: 0.5 g of jujube was added to 20, 40, 60, 80, and 100% (v/v) ethanol. The following solid‐to‐liquid (S/L) ratios were used: 1:10, 1:30, 1:50, 1:70, and 1:90 g/ml; the temperatures were: 20, 35 50, 65, and 80°C; extraction time was: 10, 30, 50, 70, and 90 min; ultrasonic power was: 100, 250, 400, 550, and 700 W. A group without sample addition was defined as a control group and was also prepared at the same time.

### 2.4 Experimental designs

#### 2.4.1 Single‐factor investigation

Variance analysis was conducted to evaluate five extraction variables: (1) extraction time, (2) extraction solvent concentration, (3) solid‐to‐liquid (S/L) ratio, (4) ultrasound power, and (5) temperature. There were four central points in this model. The main effects of the variables on the TSC were determined by calculating the difference between the lower and higher levels of the measured mean. The t‐test was used to examine the significance of the effect of each variable on the TSC.

#### 2.4.2 Response surface methodology

Single‐factor experiments indicated that extraction time, ultrasonic power, solution concentration, and solidto‐liquid (S/L) ratio had a significant effect on the extraction rate of antioxidant components; yet, this needed further optimization. Thus, a four‐factor five‐level CCD was run 30 times to determine the optimal values of important extraction factors and their effects on TSC, TFC, TPC, and antioxidant activity (%ABTSsc, %‐OH sc, %PAsc, and FRAP). The tested variables were: extraction time X_1_, ultrasonic power X_2_, solvent concentration X_3_, and solid‐to‐liquid ratio (S/L ratio) X_4_. Each variable was examined at five different levels, including factor point (−1, +1), axial points (−2, +2), and the central point (0) (Table 1).

**TABLE 1:** Experimental factors of central composite design (CCD) for extraction of sour jujube

Variable	Levels				
	−2	−1	0	1	2
X1 = extraction time (min)	10	30	50	70	90
X2 = the power of ultrasound (w)	100	250	400	550	700
X3 = percentage of solvent in the water (%)	20	40	60	80	100
X4 = solid:liquid ratio (%)	1:10	1:25	1:40	1:55	1:70

#### 2.4.3 Total saponin content

The Total saponin content (TSC) of the extracts was determined by the jujuboside A reagent method (Tang et al., 2017). A six‐point standard curve (0.0359–0.8975 mg) was constructed using jujuboside A as the reference standard, and the TSC values (mg/g) per gram of dry extract were calculated according to the following formula:

(1) $$\mathrm{TSC}\;\left(\mathrm{mg}/g\right)=\mathrm{ncv}/\left(m\times 1000\right)$$

where C (mg/g) is the total content of saponin compounds (mg/g), C is the concentration of jujuboside A obtained established from the calibration curve (mg), V is the volume of ethanol or methanol used (ml), m is the weight of sour jujube (g), and n is the dilution ratio.

#### 2.4.4 Total flavonoid content

The Total flavonoid content (TFC) of the extracts was determined by the rutin reagent method (Fan et al., 2015). A five‐point standard curve (0.0602–1.204 mg/ml) was constructed using rutin as the reference standard, and the TFC values (mg/g) per gram of dry extract were calculated according to the following formula:

(2) $$\mathrm{TFC}\;\left(\mathrm{mg}/g\right)=\mathrm{ncv}/\left(m\times 1000\right)$$

where C (mg/g) is the total content of flavonoid compounds (mg/g), and C, V, and m are the same as defined for TSC determination.

#### 2.4.5 Total phenolic content

The Total phenolic content (TPC) of the extracts was determined by the Folin–Ciocalteu method (Oroian et al., 2020). A five‐point standard curve (0.042–0.1344 mg/ml) was constructed using ferulic acid as the reference standard, and the TPC values (mg/g) per gram of dry extract were calculated according to the following formula:

(3) $$\mathrm{TPC}\;\left(\mathrm{mg}/g\right)=\mathrm{ncv}/\left(m\times 1000\right)$$

where C (mg/g) is the total content of phenolic compounds (mg/g), and C, V, and m are as defined for TSC determination.

#### 2.4.6 Antioxidant activity

##### Hydroxyl radical (•OH) scavenging assay

OH^−^ was determined based on the salicylic acid method as previously described (Sforcin, 2016). A total of 1 ml of FeSO_4_ solution (6 mmol/L), H_2_O_2_ solution (6 mmol/L), and salicylic acid ethanol solution (6 mmol/L) were added to the sample or 1 ml of the positive control vitamin C (0.8 mg/ml). After mixing well, the samples were placed in a water bath at 37°C for 1 h, and the absorbance was measured at 510 nm. The blank contained 1 ml of distilled water, the control contained no distilled water, and the other steps were the same as described above. The –OH scavenging capacity of the extracts was calculated according to Formula 4:

(4) $$\%-\mathrm{OH}\;\mathrm{sc}=\left[\left(\text{Abssample}-\text{Abscontrol}\right)/\left(\text{Abscontrol}-\text{Absblank}\right)\right]\times 100\%$$

##### Total reduction ability assay

The total reduction capacity was determined by the potassium ferric cyanide reduction method, also known as the ferric‐reducing antioxidant power (FRAP) (Pradal et al., 2016). A total of 2.5 ml of phosphate buffer solution (pH = 6.6) and 2.5 ml of potassium ferricyanide solution (1%, w/v) were added to the sample or 1 ml of the positive control vitamin C (0.8 mg/ml), respectively. After mixing, the samples were placed in a water bath at 50°C for 20 min. After cooling, 2 ml of trichloroacetic acid (10%, w/v) was added and mixed. After centrifugation (90 *g*) for 10 min, 2.5 ml of supernatant was collected, 2.5 ml of anhydrous ethanol and 0.5 ml of FeCl_3_ (10%, w/v) were added, and the absorbance was measured at 700 nm. Distilled water was used as the blank control. The inhibition rate of FRAP activity was calculated according to Formula 5:

(5) $$\left(\mathrm{mM}/L\right)\text{FRAP}=\mathrm{ncv}/m$$

##### DPPH ( 1,1‐diphenyl‐2‐picrylhydrazyl) radical‐scavenging activity

The DPPH radical‐scavenging activity was determined according to the previously described approach (Fanali et al., 2018). The sample or 1 ml of the positive control vitamin C (0.8 mg/ml) was mixed with an ethanolic DPPH solution (0.25 mmol/ml) and 3 ml of distilled water, and the absorbance was determined at 517 nm after 30 min of darkness. One milliliter of distilled water was taken as the blank solution. At the same time, 1 ml of ethanolic DPPH solution was mixed with 3 ml of distilled water as a control. The DPPH radical‐scavenging rate was calculated according to Formula 6:

(6) $$\%\text{DPPH}=\left[\text{Absblank}-\left(\text{Abssample}-\text{Abscontrol}\right)/\text{Absblank}\right]\times 100\%$$

##### Total antioxidant ability assay

According to the method described by Prasad et al., the total antioxidant activity was tested by the phosphorus molybdenum method (PA) (de Groot & Rauen, 1998). A mixed solution of trisodium phosphate (10.65 mg/ml), ammonium molybdate (4.94 mg/ml), and concentrated sulfuric acid (0.0328 v/v) was prepared. The test substance or 1 ml of the positive control vitamin C (0.8 mg/ml) was mixed with 3 ml of the mixed solution. After placing the samples in a water bath at 95°C for 1.5 h, the absorbance was measured at 695 nm. Distilled water was used as the blank control. The total antioxidant capacity was calculated according to Formula 7:

(7) $$\left(\mathrm{mM}\right)\text{Total antioxidant capacity}=\mathrm{ncv}/m$$

##### H_2_O_2_ oxidative stress

A total of 720 male and female *Drosophila* were collected and randomly divided into control group, and 1%, 0.5%, and 0.1% jujube extract treatment groups. Each group included 6 tubes. The *Drosophila* in the jujube extract concentration group were fed with different doses of extract, and the control group was fed with the basal medium. The medium was changed on the fifth day. After 30 days of normal administration, *Drosophila* was moved to another empty tube, adapted to starvation for 2 h, and then moved to a clean, sterile culture test tube. At the bottom of each test, a round filter paper was immersed in a 30% H_2_O_2_ solution containing 6% glucose. The number of deaths of fruit flies was recorded every 2 h until all *Drosophila* died. The lifespan curve of each group of *Drosophila* was plotted.

### 2.5 Statistical analysis

Response surface data were analyzed using Design‐Expert software to obtain optimization process results. The model was also determined to be valid by a Design Expert. The Pearson correlation coefficient was calculated using SPSS software. A *p* value <.05 was considered to be statistically significant.

## 3 RESULTS

### 3.1 Screening significant variables through Central Composite Design

Central composite design (CCD) was used to describe the effect of ultrasonic power, extraction time, S/L ratio, and solvent concentration on the TPC, TFC, TSC, and four antioxidant activity assays. The optimal levels of these four extraction variables were also further investigated by this method.

### 3.2 CCD model fitting and statistical analysis

#### 3.2.1 Model fitting and evaluation

The results were analyzed by fitting a second‐order polynomial model, and the regression coefficients for linear, interaction, and quadratic terms were determined by employing the least‐squares method. Analysis of variance (ANOVA) was used to determine an adequate and significant matrix. The coefficient of determination (R^2^) was used to provide additional information about model fitness. The R^2^ values for TSC, TFC, TPC, %DPPHsc, FRAP, PA, and %OH − sc with methanol and ethanol extraction were 90.0%, 90.1%, 92.3%, 95.2%, 95.7%, 98.1%, 92.1%, and 90.4%, 91.2%, 92.3%, 95.8%, 97.8%, 98.6%, and 91.5%, respectively, which suggests that the model fits the results well. The p‐value for the lack of fit was not significant (*p* > .05), which confirms the validity of the obtained model.

##### Extraction using ethanol as solvent

The experimental results of TSC, TFC, TPC, %DPPHsc, FRAP, PA, and %OH−sc of the extraction solvent (ethanol or methanol) sour jujube extraction methods are shown in Tables 2 and 3. Ethanol extraction resulted in a TSC of 77.7–138.5 mg/g; a TFC of 10.5–27.0 mg/g; a TPC of 6.6–29.1 mg/g; a %DPPHsc of 4.01%–93.37%; a FRAP of 0.55–2.69 mmol/L; a PA of 0.05–0.64 mmol; a %OH–sc of 3.27%–41.63%. The highest TSC values were the following experimental conditions: X_1_ = 70.7 min, X_2_ = 540.6 W, X_3_ = 80.7%, and X_4_ = 1:70.0 g/ml; the highest extraction method for TFC was: X_1_ = 90.0 min, X_2_ = 448.5 W, X_3_ = 49.5%, and X_4_ = 1:22.1 g/ml; the highest extraction method for TPC was: X_1_ = 76.3 min, X_2_ = 505.8 W, X_3_ = 45.0%, and X_4_ = 1:51.7 g/ml.

**TABLE 2:** Central composite design (CCD) for the observed responses and predicted values for total saponin content (TSC), total flavonoid content (TFC), and total phenolic content (TPC)

Run	Coded variable levels				Observed (Y1)						Predicted (Y0)					
	X_1_	X_2_	X_3_	X_4_	TFC (mg/g)		TSC (mg/g)		TPC (mg/g)		TFC (mg/g)		TSC (mg/g)		TPC (mg/g)	
					M	E	M	E	M	E	M	E	M	E	M	E
1	−1	1	1	1	15.1	16.5	99.1	114.4	15.5	11.5	13.4	16.4	100.0	120.9	14.6	10.9
2	1	−1	1	1	14.9	12.1	122.6	115.3	17.1	15.0	14.2	14.0	118.6	116.3	16.1	15.6
3	−1	−1	−1	−1	14.5	23.1	120.5	128.8	21.3	20.4	12.9	23.1	127.5	127.8	20.5	20.4
4	0	0	0	−2	14.6	18.8	88.6	89.7	15.5	17.2	15.9	19.8	88.9	93.3	15.1	17.7
5	0	0	0	0	22.3	22.5	121.5	125.3	21.6	24.5	21.6	22.3	123.7	129.2	21.3	25.4
6	1	−1	1	−1	17.6	13.3	106.0	88.3	15.8	21.3	16.2	14.8	105.8	91.3	16.8	18.7
7	1	−1	−1	1	21.8	23.1	122.1	128.7	20.0	21.3	21.5	22.9	122.8	119.9	20.2	22.3
8	−1	1	1	−1	16.8	14.6	96.3	97.3	14.5	15.6	16.3	14.7	96.3	99.4	14.8	13.8
9	1	−1	−1	−1	15.6	23.8	100.1	113.8	18.3	18.5	16.1	24.3	97.8	108.6	19.0	19.4
10	2	0	0	0	21.5	24.9	102.3	101.5	25.3	23.4	22.7	24.1	108.6	106.8	23.7	24.6
11	0	0	0	0	23.3	21.8	122.0	131.3	20.2	26.8	21.6	22.3	123.7	129.2	21.3	25.4
12	1	1	1	−1	19.5	17.4	111.1	94.7	17.4	16.9	18.9	17.2	109.3	88.8	17.6	16.6
13	−1	1	−1	−1	14.7	23.4	91.7	97.4	16.5	18.9	14.1	21.9	94.3	97.7	17.3	18.6
14	−1	−1	−1	1	19.0	23.6	145.1	113.3	21.1	19.2	18.4	24.2	145.5	120.6	20.6	19.8
15	0	0	2	0	10.1	10.5	100.8	95.2	11.0	6.6	10.9	8.4	106.9	97.6	11.2	8.4
16	0	0	0	0	19.0	20.8	133.3	125.1	21.0	24.6	21.6	22.3	123.7	129.2	21.3	25.4
17	0	0	0	0	20.8	22.7	124.3	129.4	22.4	27.0	21.6	22.3	123.7	129.2	21.3	25.4
18	0	2	0	0	17.7	16.1	94.9	97.9	19.9	17.6	19.1	18.7	96.0	98.6	18.8	19.3
19	0	0	0	2	17.5	21.3	117.2	124.3	15.9	17.7	18.4	20.1	117.6	126.1	16.0	17.7
20	0	−2	0	0	15.5	20.2	128.4	111.6	18.7	20.6	16.3	17.4	128.0	116.3	19.5	19.4
21	0	0	−2	0	14.5	22.7	114.3	96.4	18.3	21.2	15.9	24.6	108.9	99.5	17.8	19.9
22	−1	−1	1	1	14.6	14.2	109.5	112.8	14.6	12.9	14.6	16.0	112.5	106.8	14.9	11.9
23	−1	−1	1	−1	16.0	14.5	111.0	105.3	17.9	16.1	16.5	14.3	106.6	100.2	16.7	18.5
24	1	1	−1	−1	21.0	27.0	80.6	77.7	18.4	20.0	20.1	25.1	78.3	76.9	18.6	20.3
25	−1	1	−1	1	18.1	24.7	109.1	115.1	19.4	20.0	18.6	23.1	110.0	105.4	19.0	21.8
26	1	1	−1	1	26.4	23.1	98.1	96.7	20.4	29.1	24.6	23.7	101.1	103.1	21.4	27.0
27	1	1	1	1	15.1	16.5	126.3	134.4	17.1	18.0	15.9	16.3	120.0	128.7	18.5	17.3
28	0	0	0	0	21.5	23.7	110.3	125.4	21.0	23.0	21.6	22.3	123.7	129.2	21.3	25.4
29	0	0	0	0	22.5	22.3	130.9	138.5	21.3	26.4	21.6	22.3	123.7	129.2	21.3	25.4
30	−2	0	0	0	16.0	22.3	124.0	117.9	19.9	19.9	17.0	22.9	118.4	118.0	21.2	19.2

**TABLE 3:** Central composite design (CCD) for the observed responses and predicted values of ferric‐reducing antioxidant power (FRAP), phosphorus molybdenum method (PA) and inhibition for %DPPHsc and %OH−sc

Run	Coded variable levels				Observed (Y1)								Predicted (Y0)							
	X_1_	X_2_	X_3_	X_4_	DPPH (%)		FRAP (mmol/L)		PA (mmol)		OH^−^ (%)		DPPH (%)		FRAP (mmol/L)		PA (mmol)		OH^−^ (%)	
					M	E	M	E	M	E	M	E	M	E	M	E	M	E	M	E
1	−1	1	1	1	83.83	79.38	1.46	0.97	0.27	0.05	9.07	17.25	86.61	74.70	1.52	0.88	0.28	0.04	11.65	14.48
2	1	−1	1	1	81.75	79.67	1.65	1.09	0.29	0.10	2.12	4.85	84.56	82.53	1.70	1.03	0.25	0.10	3.74	0.86
3	−1	−1	−1	−1	20.03	19.73	1.81	1.76	0.73	0.37	10.33	11.67	24.20	19.79	1.93	1.74	0.76	0.38	12.38	11.09
4	0	0	0	−2	35.01	4.01	2.13	2.23	1.34	0.64	36.72	41.63	34.89	8.59	2.12	2.20	1.30	0.62	34.61	39.20
5	0	0	0	0	68.99	72.11	1.24	1.36	0.34	0.22	8.60	19.88	65.73	67.24	1.18	1.34	0.41	0.20	19.72	29.23
6	1	−1	1	−1	73.89	63.04	1.52	1.13	0.66	0.29	33.57	27.26	73.87	60.32	1.58	1.20	0.69	0.29	34.45	31.35
7	1	−1	−1	1	47.76	54.90	1.37	0.82	0.34	0.13	3.53	5.52	51.91	56.17	1.47	0.77	0.33	0.12	2.84	6.90
8	−1	1	1	−1	76.11	75.07	2.51	1.80	0.61	0.28	7.44	13.79	72.23	74.09	2.41	1.89	0.63	0.28	6.85	16.74
9	1	−1	−1	−1	33.32	9.79	1.93	1.31	0.81	0.27	38.29	35.47	29.93	11.55	1.82	1.27	0.80	0.29	39.60	37.37
10	2	0	0	0	69.88	71.96	1.34	0.79	0.33	0.16	18.76	23.80	71.58	72.27	1.33	0.79	0.38	0.16	18.81	22.42
11	0	0	0	0	68.55	76.11	1.11	1.30	0.42	0.22	21.10	35.92	65.73	67.24	1.18	1.34	0.41	0.20	19.72	29.23
12	1	1	1	−1	72.40	73.15	1.72	1.62	0.63	0.26	26.94	39.90	76.88	71.76	1.76	1.56	0.62	0.28	28.45	36.48
13	−1	1	−1	−1	43.32	58.90	2.99	2.69	0.66	0.35	4.68	3.46	39.89	53.13	2.89	2.63	0.69	0.37	6.94	6.58
14	−1	−1	−1	1	62.02	56.33	1.74	1.08	0.31	0.14	3.46	4.11	56.92	54.81	1.65	1.01	0.30	0.14	5.83	6.65
15	0	0	2	0	85.61	74.93	1.81	1.63	0.29	0.06	8.52	8.08	80.54	75.64	1.79	1.63	0.27	0.06	7.78	7.87
16	0	0	0	0	68.55	60.39	0.94	1.28	0.43	0.20	17.64	26.26	65.73	67.24	1.18	1.34	0.41	0.20	19.72	29.23
17	0	0	0	0	56.23	74.33	1.23	1.46	0.47	0.19	25.59	31.37	65.73	67.24	1.18	1.34	0.41	0.20	19.72	29.23
18	0	2	0	0	72.31	93.37	1.25	1.30	0.33	0.17	17.57	23.09	77.91	99.48	1.39	1.31	0.34	0.17	18.13	21.64
19	0	0	0	2	70.77	55.79	0.82	0.55	0.45	0.19	3.15	7.50	71.25	53.83	0.88	0.68	0.48	0.20	2.65	6.47
20	0	−2	0	0	71.51	70.18	1.42	0.59	0.41	0.19	27.45	20.82	66.26	66.69	1.33	0.67	0.41	0.18	24.28	18.81
21	0	0	−2	0	10.12	26.42	1.97	2.01	0.38	0.17	8.84	7.00	15.55	28.33	2.04	2.11	0.40	0.17	6.97	3.76
22	−1	−1	1	1	80.42	67.12	1.51	1.04	0.22	0.07	12.83	6.03	83.33	69.96	1.48	1.02	0.25	0.08	13.19	9.13
23	−1	−1	1	−1	57.27	57.57	1.26	1.49	0.67	0.36	12.64	14.56	61.89	57.36	1.29	1.42	0.68	0.35	13.69	13.59
24	1	1	−1	−1	40.95	42.14	1.81	2.00	0.72	0.35	36.63	33.61	38.30	39.59	1.84	2.05	0.70	0.33	35.00	34.85
25	−1	1	−1	1	65.28	73.15	1.59	1.32	0.32	0.16	7.85	4.11	65.56	76.16	1.53	1.28	0.30	0.15	5.70	4.35
26	1	1	−1	1	58.46	74.93	0.49	1.00	0.32	0.16	0.71	6.48	53.22	72.23	0.41	0.94	0.30	0.18	3.54	6.58
27	1	1	1	1	84.42	81.75	0.91	0.73	0.26	0.12	6.35	3.27	80.52	81.98	0.79	0.78	0.26	0.11	3.03	8.19
28	0	0	0	0	71.36	65.88	1.50	1.21	0.35	0.17	27.20	23.73	65.73	67.24	1.18	1.34	0.41	0.20	19.72	29.23
29	0	0	0	0	60.68	54.60	1.06	1.44	0.45	0.19	18.16	38.23	65.73	67.24	1.18	1.34	0.41	0.20	19.72	29.23
30	−2	0	0	0	73.29	70.92	2.10	1.26	0.41	0.18	2.86	4.50	71.94	73.23	2.16	1.35	0.36	0.18	0.20	2.42

The optimal extraction variables of these different bioactive groups with ethanol in the highest % DPPHsc, FRAP, PA, and %OH − sc were X_1_ = 84.6, 20.4, 10.0, and 90.0 min; X_2_ = 166.3, 555.1, 57.7, and 54.6 W; X_3_ = 94.3%, 24.0%, 57.7%, and 54.6%; and X_4_ = 1:41.2, 1:11.3, 1:10.0, and 1:10.0 g/ml, respectively. The quadratic regression coefficients of X_2_, X_4_, X_1_X_4_, X_2_X_3_, X_2_X_4_, $X_{1}^{2}$, $X_{2}^{2}$, $X_{3}^{2}$, and $X_{4}^{2}$ in TSC; X_3_, $X_{2}^{2}$, $X_{3}^{2}$, and $X_{4}^{2}$ in TFC; X_1_, X_3_, X_3_X_4_, $X_{1}^{2}$, $X_{2}^{2}$, $X_{3}^{2}$, and $X_{4}^{2}$ in TPC; X_2_, X_3_, X_4_, X_2_X_3_, X_3_X_4_, $X_{2}^{2}$, $X_{3}^{2}$, and $X_{4}^{2}$ in %DPPHsc; X_1_, X_2_, X_3_, X_4_, X_1_X_3_, X_1_X_4_, X_2_X_3_, X_2_X_4_, X_3_X_4_, $X_{1}^{2}$, $X_{2}^{2}$, and $X_{3}^{2}$ in FRAP; X_3_, X_4_, X_1_X_2_, X_1_X_4_, X_2_X_3_, $X_{3}^{2}$, and $X_{4}^{2}$ in PA; and X_1_, X_4_, X_1_X_4_, $X_{1}^{2}$, $X_{2}^{2}$, and $X_{3}^{2}$ in %OH−sc were significant. The predictive model results are shown in Formula (8), (9), (10), (11), (12), (13), (14) below.
(8)

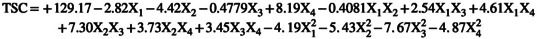

(9) $$\begin{array}{c} \mathrm{TFC}=+22.30+0.2875X_{1}+0.3042X_{2}-4.05X_{3}+0.0708X_{4}+0.4938X_{1}X_{2}-0.1688X_{1}X_{3}-0.6312X_{1}X_{4}+0.3938X_{2}X_{3}\\ \;+0.0063X_{2}X_{4}+0.1438X_{3}X_{4}+0.2969{X_{1}}^{2}-1.07{X_{2}}^{2}-1.45{X_{3}}^{2}-0.5906{X_{4}}^{2} \end{array}$$

(10) $$\begin{array}{c} \mathrm{TPC}=+25.38+1.35X_{1}-0.0292X_{2}-2.89X_{3}+0.0125X_{4}+0.6563X_{1}X_{2}+0.2938X_{1}X_{3}+0.8813X_{1}X_{4}\\ \;-0.7437X_{2}X_{3}+0.9438X_{2}X_{4}-1.52X_{3}X_{4}-0.8677{X_{1}}^{2}-1.51{X_{2}}^{2}-2.81{X_{3}}^{2}-1.92{X_{4}}^{2} \end{array}$$

(11) $$\begin{array}{c} \%\text{DPPHsc}=+67.24-0.2417X_{1}+8.20X_{2}+11.83X_{3}+11.31X_{4}-1.32X_{1}X_{2}+2.80X_{1}X_{3}+2.40X_{1}X_{4}\\ \;-4.15X_{2}X_{3}-3.00X_{2}X_{4}-5.60X_{3}X_{4}+1.38{X_{1}}^{2}+3.96{X_{2}}^{2}-3.81{X_{3}}^{2}-9.01{X_{4}}^{2} \end{array}$$

(12) $$\begin{array}{c} \text{FRAP}=+1.34-0.1413X_{1}+0.1596X_{2}-0.1196X_{3}-0.3796X_{4}-0.0256X_{1}X_{2}+0.0619X_{1}X_{3}+0.0569X_{1}X_{4}\\ \;-0.1044X_{2}X_{3}-0.1519X_{2}X_{4}+0.0831X_{3}X_{4}-0.0674{X_{1}}^{2}-0.0874{X_{2}}^{2}+0.1314{X_{3}}^{2}+0.0239{X_{4}}^{2} \end{array}$$

(13) $$\begin{array}{c} \mathrm{PA}=+0.1992-0.0059X_{1}-0.0019X_{2}-0.0272X_{3}-0.1038X_{4}+0.0133X_{1}X_{2}+0.0081X_{1}X_{3}+0.0182X_{1}X_{4}\\ \;-0.0137X_{2}X_{3}+0.0060X_{2}X_{4}-0.0055X_{3}X_{4}-0.0075{X_{1}}^{2}-0.0056{X_{2}}^{2}-0.0217{X_{3}}^{2}+0.0532{X_{4}}^{2} \end{array}$$

(14) $$\begin{array}{c} \%\mathrm{OH}-\mathrm{sc}=+29.23+5.00X_{1}+0.7061X_{2}+1.03X_{3}-8.18X_{4}+0.4947X_{1}X_{2}-2.13X_{1}X_{3}-6.51X_{1}X_{4}\\ \;+1.91X_{2}X_{3}+0.5507X_{2}X_{4}-0.0055X_{3}X_{4}-4.20{X_{1}}^{2}-2.25{X_{2}}^{2}-5.86{X_{3}}^{2}-1.60{X_{4}}^{2} \end{array}$$

##### Extraction using methanol as the solvent

Methanol extraction resulted in 80.6–145.1 mg/g in TSC from 10.1 to 26.4 mg/g for TFC, from 11.0 to 25.3 mg/g for TPC, from 10.12% to 85.61% for %DPPHsc, from 0.49 to 2.99 mmol/L for FRAP, from 0.22 mmol to 1.34 mmol for PA, and from 0.71% to 38.29% for %OH−sc. The highest TSC values were obtained using the following experimental conditions: X_1_ = 10.0 min, X_2_ = 100.0 W, X_3_ = 20.0%, and X_4_ = 1:55.9 g/ml; the highest extraction method for TFC was: X_1_ = 90.0 min, X_2_ = 582.3 W, X_3_ = 30.9%, and X_4_ = 1:60.2 g/ml; the highest extraction method for TPC was: X_1_ = 90.0 min, X_2_ = 587.5 W, X_3_ = 55.6%, and X_4_ = 1:47.2 g/ml.

The optimal extraction variables of these different bioactive groups with ethanol in the highest %DPPHsc, FRAP, PA, and %OH − sc were X_1_ = 90.0, 10.2, 82.9, and 90.0 min; X_2_ = 104.7, 687.1, 700.0, and 100.0 W; X_3_ = 94.4%, 20.1%, 23.1%, and 42.8%; and X_4_ = 1:28.9, 1:18.0, 1:70.0, and 1:10.0 g/ml, respectively. The quadratic regression coefficients of X_2_, X_4_, X_1_X_3_, X_2_X_3_, $X_{2}^{2}$, $X_{3}^{2}$, and $X_{4}^{2}$ in TSC; X_1_, X_2_, X_3_, X_1_X_3_, X_3_X_4_, $X_{2}^{2}$, $X_{3}^{2}$ and $X_{4}^{2}$ in TFC; X_1_, X_3_, X_1_X_2_, $X_{2}^{2}$, $X_{3}^{2}$, and $X_{4}^{2}$ in TPC; X_2_, X_3_, X_4_, $X_{3}^{2}$, and $X_{4}^{2}$ in % DPPHsc; X_1_, X_4_, X_1_X_2_, X_1_X_3_, X_2_X_4_, X_3_X_4_, $X_{1}^{2}$, $X_{3}^{2}$, and $X_{4}^{2}$ in FRAP; X_3_, X_4_, $X_{3}^{2}$, and $X_{4}^{2}$ in PA; and X_1_, X_4_, X_1_X_4_, $X_{1}^{2}$, and $X_{3}^{2}$ in %OH − sc were significant. The predictive model results are shown in Formula (15), (16), (17), (18), (19), (20), (21) below.

(15) $$\begin{array}{c} \mathrm{TSC}=+123.72-2.45X_{1}-7.98X_{2}-0.5167X_{3}+7.16X_{4}+3.45X_{1}X_{2}+7.23X_{1}X_{3}+1.75X_{1}X_{4}\\ \;+5.75X_{2}X_{3}-0.5500X_{2}X_{4}-3.02X_{3}X_{4}-2.56{X_{1}}^{2}-2.93{X_{2}}^{2}-3.96{X_{3}}^{2}-5.12{X_{4}}^{2} \end{array}$$

(16) $$\begin{array}{c} \mathrm{TFC}=+21.57+1.42X_{1}+0.7125X_{2}-1.26X_{3}+0.6292X_{4}+0.7188X_{1}X_{2}-0.8687X_{1}X_{3}-0.0187X_{1}X_{4}\\ \;-0.3687X_{2}X_{3}-0.2437X_{2}X_{4}-1.86X_{3}X_{4}-0.4344{X_{1}}^{2}-0.9719{X_{2}}^{2}-2.05{X_{3}}^{2}-1.11{X_{4}}^{2} \end{array}$$

(17) $$\begin{array}{c} \mathrm{TPC}=+21.25+0.6042X_{1}-0.1875X_{2}-1.67X_{3}+0.2458X_{4}+0.6938X_{1}X_{2}+0.3813X_{1}X_{3}+0.2688X_{1}X_{4}\\ \;+0.3188X_{2}X_{3}+0.3813X_{2}X_{4}-0.4812X_{3}X_{4}+0.2990{X_{1}}^{2}-0.5260{X_{2}}^{2}-1.69{X_{3}}^{2}-1.43{X_{4}}^{2} \end{array}$$

(18) $$\begin{array}{c} \%\text{DPPHs}=+65.73-0.0896X_{1}+2.91X_{2}+16.25X_{3}+9.09X_{4}-1.83X_{1}X_{2}+1.56X_{1}X_{3}-2.69X_{1}X_{4}\\ \;-1.34X_{2}X_{3}-1.76X_{2}X_{4}-2.82X_{3}X_{4}+1.51{X_{1}}^{2}+1.59{X_{2}}^{2}-4.42{X_{3}}^{2}-3.17{X_{4}}^{2} \end{array}$$

(19) $$\begin{array}{c} \text{FRAP}=+1.18-0.2079X_{1}+0.0146X_{2}-0.0629X_{3}-0.3104X_{4}-0.2356X_{1}X_{2}+0.0994X_{1}X_{3}-0.0181X_{1}X_{4}\\ \;+0.0394X_{2}X_{3}-0.2706X_{2}X_{4}+0.1169X_{3}X_{4}+0.1411{X_{1}}^{2}+0.0449{X_{2}}^{2}+0.1836{X_{3}}^{2}+0.0799{X_{4}}^{2} \end{array}$$

(20) $$\begin{array}{c} \mathrm{PA}=+0.4092+0.0042X_{1}-0.0171X_{2}-0.0324X_{3}-0.2061X_{4}-0.0057X_{1}X_{2}-0.0063X_{1}X_{3}-0.0038X_{1}X_{4}\\ \;+0.0067X_{2}X_{3}+0.0175X_{2}X_{4}+0.0079X_{3}X_{4}-0.0102{X_{1}}^{2}-0.0096{X_{2}}^{2}-0.0196{X_{3}}^{2}+0.1208{X_{4}}^{2} \end{array}$$

(21) $$\begin{array}{c} \%\mathrm{OH}-\mathrm{sc}=+19.72+4.65X_{1}-1.54X_{2}+0.2017X_{3}-7.99X_{4}-0.2088X_{1}X_{2}-1.62X_{1}X_{3}-7.55X_{1}X_{4}\\ \;-0.3513X_{2}X_{3}+1.32X_{2}X_{4}+1.51X_{3}X_{4}-2.55{X_{1}}^{2}+0.3727{X_{2}}^{2}-3.08{X_{3}}^{2}-0.2710{X_{4}}^{2} \end{array}$$

By optimizing the extraction conditions, under various factors, the TFC and TPC extracted by ethanol were higher than those extracted by methanol, while the TSC extracted by methanol was higher.

By optimizing the extraction conditions, under the influence of various factors, the DPPH radical‐scavenging capacity, total antioxidant capacity, and total reducing capacity of the methanol extract were higher than those of the ethanol extract, while the hydroxyl‐scavenging capacity of the ethanol extract was more optimal.

#### 3.2.2 Verification of the predictive models

The optimized extraction process for obtaining maximum values of TSC, TFC, TPC, and antioxidant activities (%DPPHsc, FRAP, PA, and %OH–sc) was obtained based on the analysis of the results of the response surface methodology study. The optimal extraction process results are shown in Table 4.

**TABLE 4:** Observed and predicted values under optimum extraction conditions with ethanol and methanol as the extraction solvents

Methods	Extraction variables‐Ethanol				Obs (Y1)	Pred (Y0)	Methods	Extraction variables‐Methanol				Obs (Y1)	Pred (Y0)
	X1 (min)	X2 (W)	X3 (%)	X4 (%)				X1 (min)	X2 (W)	X3 (%)	X4 (%)		
TFC (mg/g)	90.0	448.5	49.5	1:22.1	22.3	28.3	TFC (mg/g)	90.0	582.3	30.9	1:60.2	24.0	26.6
	90.0	448.5	49.5	1:22.1	21.2	28.3		90.0	582.3	30.9	1:60.2	19.3	26.6
	90.0	448.5	49.5	1:22.1	20.2	28.3		90.0	582.3	30.9	1:60.2	21.0	26.6
TSC (mg/g)	70.7	540.6	80.7	1:70.0	126.7	134.2	TSC (mg/g)	10.0	100.0	20.0	1:55.9	128.1	179.2
	70.7	540.6	80.7	1:70.0	117.8	134.2		10.0	100.0	20.0	1:55.9	109.5	179.2
	70.7	540.6	80.7	1:70.0	111.8	134.2		10.0	100.0	20.0	1:55.9	97.5	179.2
TPC (mg/g)	76.3	505.8	45.0	1:51.7	26.6	27.3	TPC (mg/g)	90.0	587.5	55.6	1:47.2	23.1	24.7
	76.3	505.8	45.0	1:51.7	21.0	27.3		90.0	587.5	55.6	1:47.2	19.3	24.7
	76.3	505.8	45.0	1:51.7	30.1	27.3		90.0	587.5	55.6	1:47.2	29.3	24.7
DPPH (%)	84.6	166.3	94.3	1:41.2	62.91	107.6	DPPH (%)	90.0	104.7	94.4	1:28.9	79.67	100.6
	84.6	166.3	94.3	1:41.2	56.97	107.6		90.0	104.7	94.4	1:28.9	81.16	100.6
	84.6	166.3	94.3	1:41.2	58.16	107.6		90.0	104.7	94.4	1:28.9	82.20	100.6
FRAP (mmol/L)	20.4	555.1	24.0	1:11.3	2.53	1.48	FRAP (mmol/L)	10.2	687.1	20.1	1:18.0	1.63	1.37
	20.4	555.1	24.0	1:11.3	2.50	1.48		10.2	687.1	20.1	1:18.0	1.34	1.37
	20.4	555.1	24.0	1:11.3	2.46	1.48		10.2	687.1	20.1	1:18.0	1.52	1.37
PA (mmol)	10.0	100.0	57.7	1:10.0	1.66	0.73	PA (mmol)	82.9	700.0	23.1	1:70.0	0.34	1.45
	10.0	100.0	57.7	1:10.0	1.63	0.73		82.9	700.0	23.1	1:70.0	0.30	1.45
	10.0	100.0	57.7	1:10.0	1.56	0.73		82.9	700.0	23.1	1:70.0	0.35	1.45
OH^−^ (%)	90.0	402.6	54.6	1:10.0	90.76	58.86	OH^−^ (%)	90.0	100.0	42.8	1:10.0	73.00	75.25
	90.0	402.6	54.6	1:10.0	91.34	58.86		90.0	100.0	42.8	1:10.0	72.16	75.25
	90.0	402.6	54.6	1:10.0	88.58	58.86		90.0	100.0	42.8	1:10.0	72.48	75.25

Abbreviations: Obs, observed; Pred, predicted.

### 3.3 Levels of TPC, TFC, and TSC in different cultivars of sour jujube

In this experiment, the extraction conditions, namely, extraction time, ultrasonic power, solvent concentration, and S/L ratio, were optimized to obtain bioactive substances in sour jujube by ultrasound. As shown in Table 5, slight changes in TPC content were found among different producing areas. The TPC of Yangquan (Shanxi Province) was 20.411 mg/g, and that of Hotan (Xinjiang Province) was 10.293 mg/g. The order of TFC content was: Yangquan (Shanxi) > Linyi (Shandong) > Lvliang (Shanxi) > Chaoyang (Liaoning) > Dunhuang (Gansu) > Hotan (Xinjiang). Lvliang (Shanxi) samples had the maximum TSC content, followed by Dunhuang (Gansu) samples, and the TFC content of Yangquan (Shanxi) was relatively low.

**TABLE 5:** The effect of ethanol on the total flavonoid content (TFC), total phenolic content (TPC), total saponin content (TSC), and total antioxidant activity of different cultivars of sour jujube

Sample	TSC (mg/g)	TFC (mg/g)	TPC (mg/g)	DPPH (%)	FRAP (mmol/L)	PA (mmol)	OH^−^ (%)
Yangquan (Shanxi)	124.1	21.6	20.4	72.96	3.01	0.56	93.65
Hotan (Xinjiang)	181.9	6.6	10.3	74.96	1.00	0.73	35.48
Lvliang (Shanxi)	270.4	10.5	15.5	73.74	1.64	1.08	72.46
Dunhuang (Gansu)	243.2	8.6	14.0	75.89	1.22	0.96	66.46
Linyi (Shandong)	176.9	19.0	17.9	79.01	2.06	0.90	84.29
Chaoyang (Liaoning)	170.7	9.8	17.3	67.21	1.88	0.69	92.14

### 3.4 The levels of TPC, TFC, and TSC in different parts of sour jujube

The ultrasonic extraction of Yangquan (Shanxi) jujube flesh, shells, and seeds was carried out with ethanol as extraction solvent. The content of TPC, TFC, and TSC, as well as the biological activity, was compared. As shown in Table 6, the content of TFC, TSC, and TPC of flesh was higher than in other parts, and the antioxidant capacities (PA, FRAP, %OH, %DPPHsc) were stronger than in other parts. In terms of the content of active ingredients, the TSC of the shells and the TFC and TPC of the seeds were lower. Regarding biological activity, PA and FRAP in the shells and %OH, %DPPHsc in the seeds were lower.

**TABLE 6:** The effect of ethanol on the total flavonoid content (TFC), total phenolic content (TPC), total saponin content (TSC), and total antioxidant activity of different parts of sour jujube

Sample	TFC (mg/g)	TSC (mg/g)	TPC (mg/g)	DPPH (%)	FRAP (mmol/L)	PA (mmol)	OH^−^ (%)
Sour jujube	23.1	126.2	19.8	75.81	2.96	0.68	90.23
flesh	33.6	147.4	25.1	87.99	3.13	0.84	95.09
shell	5.5	30.9	5.5	62.52	0.55	0.06	33.64
seed	3.3	37.6	9.3	54.56	0.38	0.08	4.62

### 3.5 Effect of sour jujube extract on the life span of *Drosophila* under H_2_O_2_ oxidative stress

**FIGURE 1:**
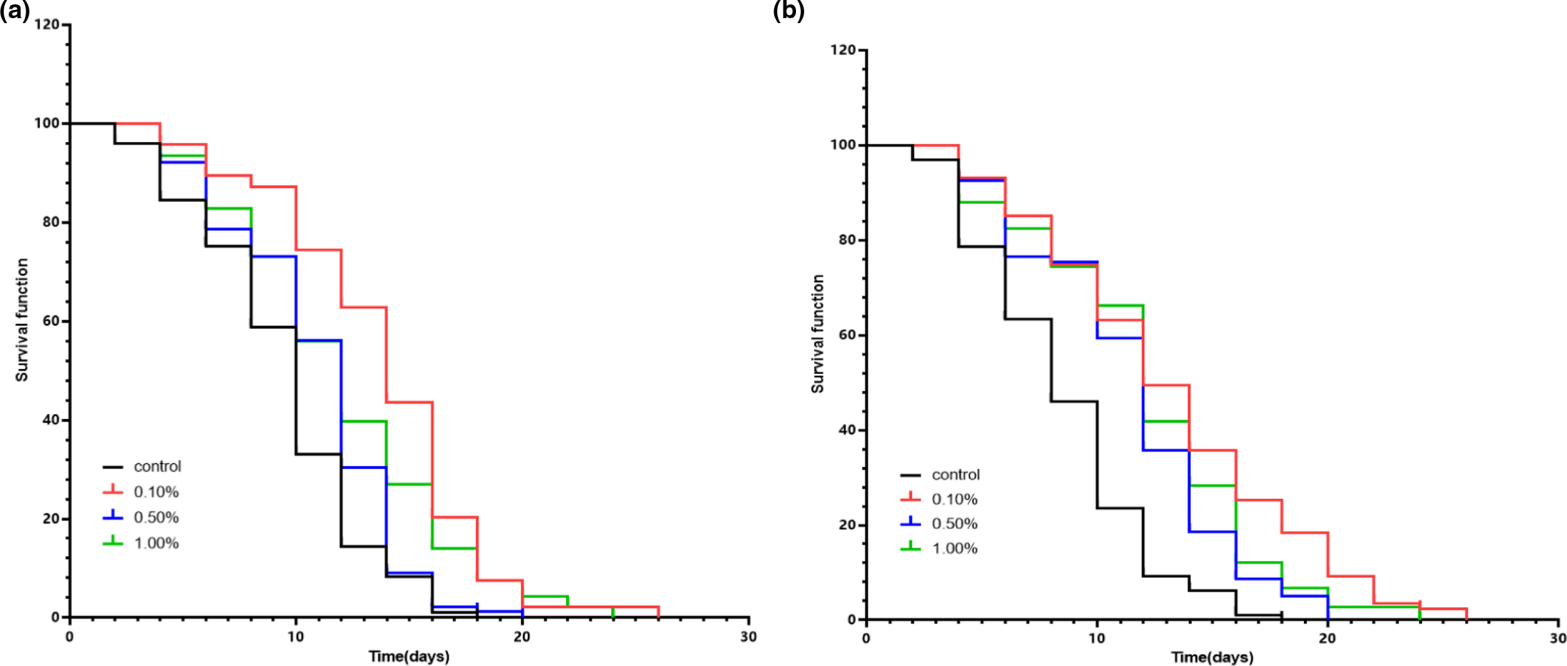
*Drosophila* lifespan curve under oxidative stress. Notes: A:male *Drosophila growth curve; B:Female Drosophila growth curve*

To study the antiaging effect of sour jujube extract, the H_2_O_2_ oxidative stress models were created to provide the condition of functions declining and damaging. As shown in Figure 1, the survival time of the different concentration treatment groups was obviously extended for both male and female *Drosophila* compared with that of the control group.

### 3.6 Analysis of sour jujube by HPLC‐UV

**FIGURE 2:**
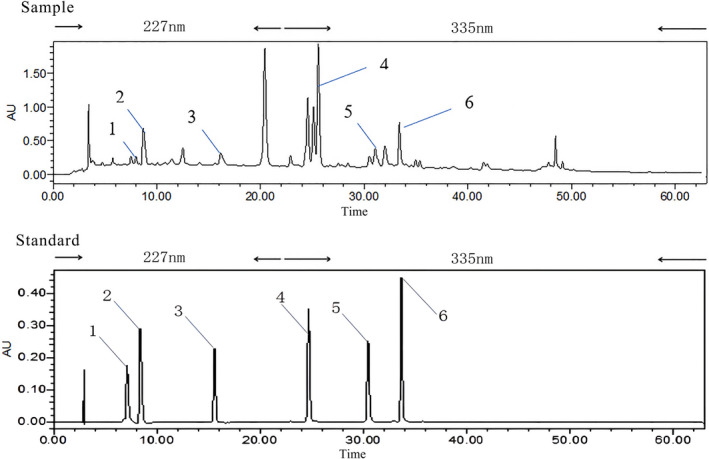
Representative high‐performance liquid chromatography‐ultraviolet (HPLC‐UV) chromatogram of sour jujube. Notes:1 coclaurine;2 vicenin;3 magnoflorine;4 spinosin;5 swertisin;6 kaempferol‐3‐O‐rutinoside;7 6'''‐feruloylspinosin

After being treated with a macroporous resin column, the concentrated sample solution was analyzed by high‐performance liquid chromatography (HPLC). Separation was achieved on a Waters Symmetry C_18_ column (250 mm × 4.6 mm, 5 μm) with a mobile phase of acetonitrile–aqueous 0.1% formic acid solution. The following gradient elution program was used: 0 ~ 26 min, 10 ~ 20% acetonitrile; 26 ~ 30 min, 20 ~ 23% acetonitrile; 30 ~ 43 min, 23 ~ 26% acetonitrile; 43 ~ 45 min, 26 ~ 37% acetonitrile; 45 ~ 47 min, 37% acetonitrile; 47 ~ 54 min, 37 ~ 39% acetonitrile; and 54 ~ 63 min, 39 ~ 100% acetonitrile. The flow rate was 1.0 ml/min, the column temperature was 25°C, the injection volume was 10 μl, and the ultraviolet (UV) detection wavelengths were 227 nm and 335 nm. A representative HPLC‐UV chromatogram is shown in Figure 2.

## 4 DISCUSSION

This study optimized the ultrasonic extraction process parameters to extract bioactive substances from sour jujube. Two extraction solvents, methanol and ethanol, were used to optimize the extraction parameters screened by CCD. The results showed that optimizing methanol and ethanol extraction process parameters could improve target components’ extraction efficiency. Under the optimum conditions, the TFC and TPC yields of sour jujube obtained by using ethanol were better than those obtained by using methanol, while the yield of TSC obtained by using methanol was better than that obtained by ethanol.

Ultrasonic‐assisted extraction is a rapid and effective extraction method that has a higher extraction rate and detection efficiency than traditional extraction methods, and provides a good solution to the selectivity of target compounds (Oroian et al., 2020; Pradal et al., 2016; Sforcin, 2016). The high TPC values obtained with ethanol in this study may be attributed to the better solvation of the phenolic compounds due to the interactions (hydrogen bonds) between the polar sites of the phenol molecules and the solvent. As observed in the phenolic content, the extraction of flavonoids was also affected by the polarity of the solvents. Therefore, increased ethanol concentration results in a greater diffusion rate of the compounds from the inside out of the plant matrix particle, which makes it a more favored approach. It is reported to be more efficient when performed using intermediate polarity solvents, resulting from binary mixtures of water and ethanol (Gasmalla et al., 2017; Yilmaz et al., 2020; Zlabur et al., 2015).

The effect of ultrasonic power on the response model showed that the greater number of cavitation bubbles and energy in the system leads to the promotion of the mass transfer, so the improvement of extraction efficiency at low power is proportional to the increase of ultrasonic power (Rouhani, 2019). Yet, a violent collapse of cavities at relatively high power dissipation levels leads to the degradation of the extracted compounds. Therefore, with the increase of ultrasonic power, the content of the extract will first increase and then decrease. At the same time, high bioactive substances and antioxidant activity were obtained by increasing the S/L ratio and extraction time. Related experiments also verified that prolonging the extraction time was conducive to extracting polyphenols (Fanali et al., 2018). Using a lower solid‐to‐solvent ratio than the optimum value leads to an increase in solvent consumption. Higher solid‐to‐solvent ratio than the optimum value will result in incomplete extraction (Palsikowski et al., 2020). With an increasing S/L ratio, more proteins and polysaccharides were dissolved in the solution, which hindered the dissolution of saponins. The antioxidant activity of many flavonoids varies greatly and is related to the chemical structure of flavonoids (de Groot & Rauen, 1998). Some studies have shown that the effect of flavonoids depends on the concentration and the source of free radicals (Lahouel et al., 2006; Palsikowski et al., 2020). In this experiment, TFC had a significant effect on the total reduction capacity, which is also consistent with the above‐indicated observations.

The TFC, TSC, and TPC yields of sour jujube from different production areas were significantly different. These differences may be related to the growth factors, such as altitude, climate, temperature, and soil moisture, which can affect the genetic relationship (Jiang et al., 2020; Karimi et al., 2020). It has been confirmed that the fruit quality and the activity of anthocyanin‐related enzymes of sour jujube can be affected by increasing temperature and drought stress (Jiang et al., 2020). Therefore, attention should be paid to the influence of production areas when purchasing and eating sour jujube.

The contents of different parts of sour jujube also significantly differed. The flesh of sour jujube contained more bioactive compounds, which were lower in the shells and seeds (Table 6). The results showed a significant relationship between TSC and total antioxidant capacity, as well as the lower yield of TSC and total antioxidant capacity in the shell. Also, a significant relationship between TFC and TPC was observed, which may be related to the structural similarity of some flavonoids and phenols (Xu et al., 2013). Zhou et al. suggested that an ultrasonic‐assisted extraction is an efficient approach for the selective extraction of flavonoids (Zhou et al., 2018). The antioxidant activity of flavonoids includes reducing the formation of and scavenging free radicals. Most ingested flavonoids are extensively degraded to various phenolic acids, some of which still possess a radical‐scavenging ability. Therefore, the absorbed flavonoids and their metabolites may display an in vivo antioxidant activity (Pietta, 2000). In addition, TPC was correlated with OH– scavenging capacity and TFC was correlated with total reducing capacity, which also provided evidence for the significant relationship between OH– scavenging capacity and total reducing capacity. Besides, TFC and TPC, OH– scavenging capacity, and total reducing capacity of the seeds were lower than those of the other parts, which also proved a significant relationship between the content of the above compounds and antioxidant capacity. Zou et al. reported that the flesh contains more flavonoids and has higher antioxidant activities than the other tissues (Zou et al., 2020). This experiment also confirmed higher TSC, TPC, and TFC content and antioxidant capacity in the jujube pulp.

There is growing evidence suggesting that oxidative stress is implicated in aging and degenerative diseases (Ma, 2014). Genetic models of oxidative stress have also demonstrated that particular antioxidants can extend life span and reduce reactive oxygen species (ROS) in specific genetic backgrounds (Vrailas‐Mortimer et al., 2012). Antioxidants, especially natural antioxidants from edible materials, can reduce the risk of aging (Xiao et al., 2011). In this study, *Drosophila* was challenged with hydrogen peroxide (H_2_O_2_), which results in a shortened life span (Vrailas‐Mortimer et al., 2011). To confirm the anti‐oxidative stress effect of the Jujube extract, *Drosophila* were exposed to oxidative stress induced by H_2_O_2_. Our data suggested that Jujube extract protected against the adverse effects of oxidative stress and improved the life span of female and male *Drosophila*, thus further verifying this effect. Furthermore, we observed that the Jujube extract had differentially protective effects against oxidative stress in males and females, which may be due to the increased sensitivity of males to oxidative stress or possible differences in metabolic rates between males and females.

Modern studies have shown that sour jujube contains many sugars, alkaloids, flavonoids, triterpenoids, and saponins, which have many health functions, such as sedation and hypnosis, antioxidation, enhancing immunity, protecting the liver, and preventing arteriosclerosis. In addition to the flavonoids, saponins, and phenols involved in this experiment, other compounds such as polysaccharides may also participate in its antioxidant effect, which should be further studied (Cao et al., 2020; Maity et al., 2011).

## 5 CONCLUSION

In this research, the parameters of extraction time, solution (ethanol or methanol) concentration, ultrasonic power, and S/L ratio, which are ultrasonic extraction variables, were optimized to achieve maximum bioactive substance contents (TSC, TFC, and TPC) and optimal antioxidant activities (DPPH, PA, FRAP, and OH–) of sour jujube. Compared to methanol, ethanol was generally more effective in extracting solvent bioactive compounds. In addition, the antioxidant activities of sour jujube in vitro were related to the TSC, TFC, and TPC. Sour jujube extract can be considered as a source of natural antioxidants, which can improve the oxidative damage caused by free radicals, and is safer than synthetic antioxidants. Therefore, greater attention should be paid to the more widespread use of sour jujube.

## FUNDING INFORMATION

This research was funded by the Science Foundation for Youth of Heilongjiang Province (LH2019H106), Heilongjiang Province Postdoctoral Research Start‐up Grant (LBH‐Q16214), Heilongjiang Provincial Science Foundation Project (H2018056), Heilongjiang University of Traditional Chinese Medicine “Excellent Innovative Talent Support Program” Project (2018RCD03), National Natural Science Foundation of China (No. 81973439), and this work was also supported by The National Natural Science Foundation of China (No. 82003974) for the development of traditional Chinese medicine.

## CONFLICT OF INTEREST

The authors declare that they have no known competing financial interests or personal relationships that could have influenced the work reported in this paper.

## ETHICS APPROVAL AND CONSENT TO PARTICIPATE

All animal studies were approved by the Animal Experimental Ethical Committee of Heilongjiang University of Chinese Medicine.

## Supporting information


Appendix S1

## Data Availability

All data generated or analyzed during this study are included in this published article (and its Appendix S1). Supplementary data to this article can be found online.
