# Supplementary material for: Optimization of ultrasound‐assisted extraction method for phytochemical compounds and antioxidant activities of sour jujube extracts
Source: Food Sci Nutr. 2022 Aug 3;10(11):3736–48. doi: 10.1002/fsn3.2971 (PMC9632212; doi:10.1002/fsn3.2971)
Supplement: Supplementary file 1 — Appendix S1 [file FSN3-10-3736-s001.docx]

**Supplementary Data**

**Optimization of ultrasound assisted extraction method for phytochemical compounds and antioxidant activities of sour jujube extracts**

Yanyan Wang^1,#^, Wan Zhao^1,#^, Yixiang Li ^1^, Hang Zhao ^2^, Xiaonan Ye ^1^, Tingli Li^1^,

Zhibin Wang ^1, *^and Lili Huang ^1, *^

^1^Department of Pharmacology of Traditional Chinese Medicine, Heilongjiang University of Chinese Medicine, No. 24, Heping Road, Harbin 150040, P. R. China

^2^ Department of pharmacy, Heilongjiang Provincial Hospital, No. 82, Zhongshan Road, Harbin 150040, P. R. China

**Table of Contents**

**1. Table S1-14， S2-8**

**2. Figure S1-S3 S9-12**

**Table S1** ANOVA of the regression model. (TSC, methanol)

| Source | Sum of Squares | df | Mean Square | F-value | p-value | significant |  |
| --- | --- | --- | --- | --- | --- | --- | --- |
| Model | 5809.53 | 14 | 414.97 | 9.80 | < 0.0001 | ** |  |
| A-A | 144.06 | 1 | 144.06 | 3.40 | 0.0850 |  |  |
| B-B | 1529.61 | 1 | 1529.61 | 36.11 | < 0.0001 | ** |  |
| C-C | 6.41 | 1 | 6.41 | 0.1512 | 0.7028 |  |  |
| D-D | 1229.80 | 1 | 1229.80 | 29.03 | < 0.0001 | ** |  |
| AB | 190.44 | 1 | 190.44 | 4.50 | 0.0511 |  |  |
| AC | 835.21 | 1 | 835.21 | 19.72 | 0.0005 | ** |  |
| AD | 49.00 | 1 | 49.00 | 1.16 | 0.2991 |  |  |
| BC | 529.00 | 1 | 529.00 | 12.49 | 0.0030 | ** |  |
| BD | 4.84 | 1 | 4.84 | 0.1143 | 0.7400 |  |  |
| CD | 146.41 | 1 | 146.41 | 3.46 | 0.0827 |  |  |
| A² | 179.23 | 1 | 179.23 | 4.23 | 0.0575 |  |  |
| B² | 235.67 | 1 | 235.67 | 5.56 | 0.0323 | * |  |
| C² | 429.31 | 1 | 429.31 | 10.13 | 0.0062 | ** |  |
| D² | 718.67 | 1 | 718.67 | 16.97 | 0.0009 | ** |  |
| Residual | 635.42 | 15 | 42.36 |  |  |  |  |
| Lack of Fit | 303.77 | 10 | 30.38 | 0.4580 | 0.8627 |  |  |
| Pure Error | 331.65 | 5 | 66.33 |  |  |  |  |
| Cor Total | 6444.95 | 29 |  |  |  |  |  |

**Table S2** ANOVA of the regression model. (TSC, ethanol)

| Source | Sum of Squares | df | Mean Square | F-value | p-value | significant |  |
| --- | --- | --- | --- | --- | --- | --- | --- |
| **Model** | 6559.79 | 14 | 468.56 | 10.07 | < 0.0001 | ** |  |
| A-A | 190.35 | 1 | 190.35 | 4.09 | 0.0613 |  |  |
| B-B | 468.26 | 1 | 468.26 | 10.07 | 0.0063 | ** |  |
| C-C | 5.48 | 1 | 5.48 | 0.1178 | 0.7362 |  |  |
| D-D | 1611.63 | 1 | 1611.63 | 34.64 | < 0.0001 | ** |  |
| AB | 2.67 | 1 | 2.67 | 0.0573 | 0.8141 |  |  |
| AC | 103.07 | 1 | 103.07 | 2.22 | 0.1573 |  |  |
| AD | 340.31 | 1 | 340.31 | 7.31 | 0.0163 | * |  |
| BC | 852.49 | 1 | 852.49 | 18.32 | 0.0007 | ** |  |
| BD | 222.08 | 1 | 222.08 | 4.77 | 0.0452 | * |  |
| CD | 190.92 | 1 | 190.92 | 4.10 | 0.0610 |  |  |
| A² | 481.61 | 1 | 481.61 | 10.35 | 0.0058 | ** |  |
| B² | 808.08 | 1 | 808.08 | 17.37 | 0.0008 | ** |  |
| C² | 1611.62 | 1 | 1611.62 | 34.64 | < 0.0001 | ** |  |
| D² | 649.27 | 1 | 649.27 | 13.96 | 0.0020 | ** |  |
| **Residual** | 697.85 | 15 | 46.52 |  |  |  |  |
| Lack of Fit | 560.45 | 10 | 56.05 | 2.04 | 0.2233 |  |  |
| Pure Error | 137.39 | 5 | 27.48 |  |  |  |  |
| **Cor Total** | 7257.64 | 29 |  |  |  |  |  |

**Table S3** ANOVA of the regression model. (TFC, methanol)

| Source | Sum of Squares | df | Mean Square | F-value | p-value | significant |  |
| --- | --- | --- | --- | --- | --- | --- | --- |
| **Model** | 330.10 | 14 | 23.58 | 9.71 | < 0.0001 | ** |  |
| A-A | 48.45 | 1 | 48.45 | 19.95 | 0.0005 | ** |  |
| B-B | 12.18 | 1 | 12.18 | 5.02 | 0.0407 | * |  |
| C-C | 38.25 | 1 | 38.25 | 15.75 | 0.0012 | ** |  |
| D-D | 9.50 | 1 | 9.50 | 3.91 | 0.0666 |  |  |
| AB | 8.27 | 1 | 8.27 | 3.40 | 0.0849 |  |  |
| AC | 12.08 | 1 | 12.08 | 4.97 | 0.0415 | * |  |
| AD | 0.0056 | 1 | 0.0056 | 0.0023 | 0.9622 |  |  |
| BC | 2.18 | 1 | 2.18 | 0.8959 | 0.3589 |  |  |
| BD | 0.9506 | 1 | 0.9506 | 0.3915 | 0.5409 |  |  |
| CD | 55.13 | 1 | 55.13 | 22.70 | 0.0003 | ** |  |
| A² | 5.18 | 1 | 5.18 | 2.13 | 0.1650 |  |  |
| B² | 25.91 | 1 | 25.91 | 10.67 | 0.0052 | ** |  |
| C² | 114.92 | 1 | 114.92 | 47.32 | < 0.0001 | ** |  |
| D² | 33.76 | 1 | 33.76 | 13.90 | 0.0020 | ** |  |
| **Residual** | 36.43 | 15 | 2.43 |  |  |  |  |
| Lack of Fit | 24.83 | 10 | 2.48 | 1.07 | 0.5009 |  |  |
| Pure Error | 11.59 | 5 | 2.32 |  |  |  |  |
| **Cor Total** | 366.52 | 29 |  |  |  |  |  |

**Table S4** ANOVA of the regression model. (TFC, ethanol)

| Source | Sum of Squares | df | Mean Square | F-value | p-value | significant |  |
| --- | --- | --- | --- | --- | --- | --- | --- |
| **Model** | 503.30 | 14 | 35.95 | 11.06 | < 0.0001 | ** |  |
| A-A | 1.98 | 1 | 1.98 | 0.6105 | 0.4467 |  |  |
| B-B | 2.22 | 1 | 2.22 | 0.6834 | 0.4214 |  |  |
| C-C | 392.85 | 1 | 392.85 | 120.91 | < 0.0001 | ** |  |
| D-D | 0.1204 | 1 | 0.1204 | 0.0371 | 0.8499 |  |  |
| AB | 3.90 | 1 | 3.90 | 1.20 | 0.2905 |  |  |
| AC | 0.4556 | 1 | 0.4556 | 0.1402 | 0.7133 |  |  |
| AD | 6.38 | 1 | 6.38 | 1.96 | 0.1816 |  |  |
| BC | 2.48 | 1 | 2.48 | 0.7635 | 0.3960 |  |  |
| BD | 0.0006 | 1 | 0.0006 | 0.0002 | 0.9891 |  |  |
| CD | 0.3306 | 1 | 0.3306 | 0.1018 | 0.7541 |  |  |
| A² | 2.42 | 1 | 2.42 | 0.7440 | 0.4020 |  |  |
| B² | 31.15 | 1 | 31.15 | 9.59 | 0.0074 | ** |  |
| C² | 57.92 | 1 | 57.92 | 17.83 | 0.0007 | ** |  |
| D² | 9.57 | 1 | 9.57 | 2.94 | 0.1067 |  |  |
| **Residual** | 48.74 | 15 | 3.25 |  |  |  |  |
| Lack of Fit | 44.08 | 10 | 4.41 | 4.73 | 0.0501 |  |  |
| Pure Error | 4.66 | 5 | 0.9320 |  |  |  |  |
| **Cor Total** | 552.04 | 29 |  |  |  |  |  |

**Table S5** ANOVA of the regression model. (TPC, methanol)

| Source | Sum of Squares | df | Mean Square | F-value | p-value | significant |  |
| --- | --- | --- | --- | --- | --- | --- | --- |
| **Model** | 229.19 | 14 | 16.37 | 12.76 | < 0.0001 | ** |  |
| A-A | 8.76 | 1 | 8.76 | 6.83 | 0.0196 | * |  |
| B-B | 0.8438 | 1 | 0.8438 | 0.6576 | 0.4301 |  |  |
| C-C | 67.00 | 1 | 67.00 | 52.22 | < 0.0001 | ** |  |
| D-D | 1.45 | 1 | 1.45 | 1.13 | 0.3045 |  |  |
| AB | 7.70 | 1 | 7.70 | 6.00 | 0.0270 | * |  |
| AC | 2.33 | 1 | 2.33 | 1.81 | 0.1982 |  |  |
| AD | 1.16 | 1 | 1.16 | 0.9007 | 0.3576 |  |  |
| BC | 1.63 | 1 | 1.63 | 1.27 | 0.2780 |  |  |
| BD | 2.33 | 1 | 2.33 | 1.81 | 0.1982 |  |  |
| CD | 3.71 | 1 | 3.71 | 2.89 | 0.1099 |  |  |
| A² | 2.45 | 1 | 2.45 | 1.91 | 0.1871 |  |  |
| B² | 7.59 | 1 | 7.59 | 5.92 | 0.0280 | * |  |
| C² | 78.20 | 1 | 78.20 | 60.95 | < 0.0001 | ** |  |
| D² | 55.78 | 1 | 55.78 | 43.47 | < 0.0001 | ** |  |
| **Residual** | 19.25 | 15 | 1.28 |  |  |  |  |
| Lack of Fit | 16.57 | 10 | 1.66 | 3.10 | 0.1120 |  |  |
| Pure Error | 2.68 | 5 | 0.5350 |  |  |  |  |
| **Cor Total** | 248.43 | 29 |  |  |  |  |  |

**Table S6** ANOVA of the regression model. (TPC, ethanol)

| Source | Sum of Squares | df | Mean Square | F-value | p-value | significant |  |
| --- | --- | --- | --- | --- | --- | --- | --- |
| **Model** | 629.81 | 14 | 44.99 | 12.90 | < 0.0001 | ** |  |
| A-A | 44.01 | 1 | 44.01 | 12.62 | 0.0029 | ** |  |
| B-B | 0.0204 | 1 | 0.0204 | 0.0059 | 0.9400 |  |  |
| C-C | 200.10 | 1 | 200.10 | 57.36 | < 0.0001 | ** |  |
| D-D | 0.0038 | 1 | 0.0038 | 0.0011 | 0.9743 |  |  |
| AB | 6.89 | 1 | 6.89 | 1.98 | 0.1803 |  |  |
| AC | 1.38 | 1 | 1.38 | 0.3958 | 0.5387 |  |  |
| AD | 12.43 | 1 | 12.43 | 3.56 | 0.0786 |  |  |
| BC | 8.85 | 1 | 8.85 | 2.54 | 0.1320 |  |  |
| BD | 14.25 | 1 | 14.25 | 4.09 | 0.0615 |  |  |
| CD | 36.91 | 1 | 36.91 | 10.58 | 0.0054 | ** |  |
| A² | 20.65 | 1 | 20.65 | 5.92 | 0.0280 | * |  |
| B² | 62.14 | 1 | 62.14 | 17.81 | 0.0007 | ** |  |
| C² | 215.84 | 1 | 215.84 | 61.87 | < 0.0001 | ** |  |
| D² | 100.87 | 1 | 100.87 | 28.92 | < 0.0001 | ** |  |
| **Residual** | 52.33 | 15 | 3.49 |  |  |  |  |
| Lack of Fit | 39.60 | 10 | 3.96 | 1.56 | 0.3269 |  |  |
| Pure Error | 12.73 | 5 | 2.55 |  |  |  |  |
| **Cor Total** | 682.14 | 29 |  |  |  |  |  |

**Table S7** ANOVA of the regression model. (DPPH, methanol)

| Source | Sum of Squares | df | Mean Square | F-value | p-value | significant |  |
| --- | --- | --- | --- | --- | --- | --- | --- |
| **Model** | 9971.84 | 14 | 712.27 | 21.06 | < 0.0001 | ** |  |
| A-A | 0.1926 | 1 | 0.1926 | 0.0057 | 0.9408 |  |  |
| B-B | 203.64 | 1 | 203.64 | 6.02 | 0.0268 | * |  |
| C-C | 6335.23 | 1 | 6335.23 | 187.34 | < 0.0001 | ** |  |
| D-D | 1983.26 | 1 | 1983.26 | 58.65 | < 0.0001 | ** |  |
| AB | 53.62 | 1 | 53.62 | 1.59 | 0.2272 |  |  |
| AC | 39.03 | 1 | 39.03 | 1.15 | 0.2996 |  |  |
| AD | 115.51 | 1 | 115.51 | 3.42 | 0.0844 |  |  |
| BC | 28.76 | 1 | 28.76 | 0.8504 | 0.3710 |  |  |
| BD | 49.81 | 1 | 49.81 | 1.47 | 0.2437 |  |  |
| CD | 127.41 | 1 | 127.41 | 3.77 | 0.0713 |  |  |
| A² | 62.43 | 1 | 62.43 | 1.85 | 0.1943 |  |  |
| B² | 69.33 | 1 | 69.33 | 2.05 | 0.1727 |  |  |
| C² | 536.18 | 1 | 536.18 | 15.86 | 0.0012 | ** |  |
| D² | 274.78 | 1 | 274.78 | 8.13 | 0.0122 | ** |  |
| **Residual** | 507.25 | 15 | 33.82 |  |  |  |  |
| Lack of Fit | 333.27 | 10 | 33.33 | 0.9578 | 0.5563 |  |  |
| Pure Error | 173.98 | 5 | 34.80 |  |  |  |  |
| **Cor Total** | 10479.08 | 29 |  |  |  |  |  |

**Table S8** ANOVA of the regression model. (DPPH, ethanol)

| Source | Sum of Squares | df | Mean Square | F-value | p-value | significant |  |
| --- | --- | --- | --- | --- | --- | --- | --- |
| **Model** | 12653.69 | 14 | 903.83 | 24.35 | < 0.0001 | ** |  |
| A-A | 1.40 | 1 | 1.40 | 0.0378 | 0.8485 |  |  |
| B-B | 1612.12 | 1 | 1612.12 | 43.43 | < 0.0001 | ** |  |
| C-C | 3358.30 | 1 | 3358.30 | 90.47 | < 0.0001 | ** |  |
| D-D | 3069.08 | 1 | 3069.08 | 82.68 | < 0.0001 | ** |  |
| AB | 28.04 | 1 | 28.04 | 0.7553 | 0.3985 |  |  |
| AC | 125.55 | 1 | 125.55 | 3.38 | 0.0858 |  |  |
| AD | 92.26 | 1 | 92.26 | 2.49 | 0.1358 |  |  |
| BC | 275.73 | 1 | 275.73 | 7.43 | 0.0156 | * |  |
| BD | 143.64 | 1 | 143.64 | 3.87 | 0.0679 |  |  |
| CD | 502.43 | 1 | 502.43 | 13.54 | 0.0022 | ** |  |
| A² | 52.12 | 1 | 52.12 | 1.40 | 0.2545 |  |  |
| B² | 430.62 | 1 | 430.62 | 11.60 | 0.0039 | ** |  |
| C² | 398.72 | 1 | 398.72 | 10.74 | 0.0051 | ** |  |
| D² | 2224.90 | 1 | 2224.90 | 59.94 | < 0.0001 | ** |  |
| **Residual** | 556.80 | 15 | 37.12 |  |  |  |  |
| Lack of Fit | 195.59 | 10 | 19.56 | 0.2708 | 0.9626 |  |  |
| Pure Error | 361.20 | 5 | 72.24 |  |  |  |  |
| **Cor Total** | 13210.48 | 29 |  |  |  |  |  |

**Table S9** ANOVA of the regression model. (FRAP, methanol)

| Source | Sum of Squares | df | Mean Square | F-value | p-value | significant |  |
| --- | --- | --- | --- | --- | --- | --- | --- |
| **Model** | 7.26 | 14 | 0.5183 | 23.81 | < 0.0001 | ** |  |
| A-A | 1.04 | 1 | 1.04 | 47.67 | < 0.0001 | ** |  |
| B-B | 0.0051 | 1 | 0.0051 | 0.2345 | 0.6352 |  |  |
| C-C | 0.0950 | 1 | 0.0950 | 4.37 | 0.0541 |  |  |
| D-D | 2.31 | 1 | 2.31 | 106.26 | < 0.0001 | ** |  |
| AB | 0.8883 | 1 | 0.8883 | 40.82 | < 0.0001 | ** |  |
| AC | 0.1580 | 1 | 0.1580 | 7.26 | 0.0166 | * |  |
| AD | 0.0053 | 1 | 0.0053 | 0.2415 | 0.6302 |  |  |
| BC | 0.0248 | 1 | 0.0248 | 1.14 | 0.3026 |  |  |
| BD | 1.17 | 1 | 1.17 | 53.84 | < 0.0001 | ** |  |
| CD | 0.2186 | 1 | 0.2186 | 10.04 | 0.0064 | ** |  |
| A² | 0.5464 | 1 | 0.5464 | 25.11 | 0.0002 | ** |  |
| B² | 0.0553 | 1 | 0.0553 | 2.54 | 0.1318 |  |  |
| C² | 0.9251 | 1 | 0.9251 | 42.51 | < 0.0001 | ** |  |
| D² | 0.1751 | 1 | 0.1751 | 8.05 | 0.0125 | * |  |
| **Residual** | 0.3264 | 15 | 0.0218 |  |  |  |  |
| Lack of Fit | 0.1410 | 10 | 0.0141 | 0.3804 | 0.9093 |  |  |
| Pure Error | 0.1854 | 5 | 0.0371 |  |  |  |  |
| **Cor Total** | 7.58 | 29 |  |  |  |  |  |

**Table S10** ANOVA of the regression model. (FRAP, ethanol)

| Source | Sum of Squares | df | Mean Square | F-value | p-value | significant |  |
| --- | --- | --- | --- | --- | --- | --- | --- |
| **Model** | 6.63 | 14 | 0.4735 | 48.09 | < 0.0001 | ** |  |
| A-A | 0.4788 | 1 | 0.4788 | 48.63 | < 0.0001 | ** |  |
| B-B | 0.6112 | 1 | 0.6112 | 62.08 | < 0.0001 | ** |  |
| C-C | 0.3432 | 1 | 0.3432 | 34.86 | < 0.0001 | ** |  |
| D-D | 3.46 | 1 | 3.46 | 351.21 | < 0.0001 | ** |  |
| AB | 0.0105 | 1 | 0.0105 | 1.07 | 0.3180 |  |  |
| AC | 0.0613 | 1 | 0.0613 | 6.22 | 0.0248 | * |  |
| AD | 0.0518 | 1 | 0.0518 | 5.26 | 0.0367 | * |  |
| BC | 0.1743 | 1 | 0.1743 | 17.70 | 0.0008 | ** |  |
| BD | 0.3691 | 1 | 0.3691 | 37.48 | < 0.0001 | ** |  |
| CD | 0.1106 | 1 | 0.1106 | 11.23 | 0.0044 | ** |  |
| A² | 0.1246 | 1 | 0.1246 | 12.65 | 0.0029 | ** |  |
| B² | 0.2095 | 1 | 0.2095 | 21.28 | 0.0003 | ** |  |
| C² | 0.4733 | 1 | 0.4733 | 48.06 | < 0.0001 | ** |  |
| D² | 0.0156 | 1 | 0.0156 | 1.59 | 0.2273 |  |  |
| **Residual** | 0.1477 | 15 | 0.0098 |  |  |  |  |
| Lack of Fit | 0.1008 | 10 | 0.0101 | 1.08 | 0.4991 |  |  |
| Pure Error | 0.0469 | 5 | 0.0094 |  |  |  |  |
| **Cor Total** | 6.78 | 29 |  |  |  |  |  |

**Table S11** ANOVA of the regression model. (Total antioxidant ability, methanol)

| Source | Sum of Squares | df | Mean Square | F-value | p-value | significant |  |
| --- | --- | --- | --- | --- | --- | --- | --- |
| **Model** | 1.52 | 14 | 0.1089 | 56.42 | < 0.0001 | ** |  |
| A-A | 0.0004 | 1 | 0.0004 | 0.2203 | 0.6456 |  |  |
| B-B | 0.0070 | 1 | 0.0070 | 3.62 | 0.0763 |  |  |
| C-C | 0.0252 | 1 | 0.0252 | 13.07 | 0.0025 | ** |  |
| D-D | 1.02 | 1 | 1.02 | 528.08 | < 0.0001 | ** |  |
| AB | 0.0005 | 1 | 0.0005 | 0.2705 | 0.6106 |  |  |
| AC | 0.0006 | 1 | 0.0006 | 0.3299 | 0.5742 |  |  |
| AD | 0.0002 | 1 | 0.0002 | 0.1190 | 0.7349 |  |  |
| BC | 0.0007 | 1 | 0.0007 | 0.3687 | 0.5528 |  |  |
| BD | 0.0049 | 1 | 0.0049 | 2.54 | 0.1321 |  |  |
| CD | 0.0010 | 1 | 0.0010 | 0.5136 | 0.4846 |  |  |
| A² | 0.0029 | 1 | 0.0029 | 1.49 | 0.2409 |  |  |
| B² | 0.0025 | 1 | 0.0025 | 1.30 | 0.2712 |  |  |
| C² | 0.0106 | 1 | 0.0106 | 5.47 | 0.0336 | * |  |
| D² | 0.4004 | 1 | 0.4004 | 207.45 | < 0.0001 | ** |  |
| **Residual** | 0.0289 | 15 | 0.0019 |  |  |  |  |
| Lack of Fit | 0.0144 | 10 | 0.0014 | 0.4975 | 0.8374 |  |  |
| Pure Error | 0.0145 | 5 | 0.0029 |  |  |  |  |
| **Cor Total** | 1.55 | 29 |  |  |  |  |  |

**Table S12** ANOVA of the regression model. (Total antioxidant ability, ethanol)

| Source | Sum of Squares | df | Mean Square | F-value | p-value | significant |  |
| --- | --- | --- | --- | --- | --- | --- | --- |
| **Model** | 0.3982 | 14 | 0.0284 | 74.01 | < 0.0001 | ** |  |
| A-A | 0.0008 | 1 | 0.0008 | 2.15 | 0.1635 |  |  |
| B-B | 0.0001 | 1 | 0.0001 | 0.2153 | 0.6493 |  |  |
| C-C | 0.0177 | 1 | 0.0177 | 46.18 | < 0.0001 | ** |  |
| D-D | 0.2588 | 1 | 0.2588 | 673.39 | < 0.0001 | ** |  |
| AB | 0.0028 | 1 | 0.0028 | 7.39 | 0.0159 | * |  |
| AC | 0.0011 | 1 | 0.0011 | 2.75 | 0.1177 |  |  |
| AD | 0.0053 | 1 | 0.0053 | 13.75 | 0.0021 | ** |  |
| BC | 0.0030 | 1 | 0.0030 | 7.86 | 0.0133 | * |  |
| BD | 0.0006 | 1 | 0.0006 | 1.51 | 0.2375 |  |  |
| CD | 0.0005 | 1 | 0.0005 | 1.25 | 0.2803 |  |  |
| A² | 0.0016 | 1 | 0.0016 | 4.06 | 0.0621 |  |  |
| B² | 0.0009 | 1 | 0.0009 | 2.26 | 0.1538 |  |  |
| C² | 0.0130 | 1 | 0.0130 | 33.70 | < 0.0001 | ** |  |
| D² | 0.0777 | 1 | 0.0777 | 202.15 | < 0.0001 | ** |  |
| **Residual** | 0.0058 | 15 | 0.0004 |  |  |  |  |
| Lack of Fit | 0.0038 | 10 | 0.0004 | 0.9464 | 0.5621 |  |  |
| Pure Error | 0.0020 | 5 | 0.0004 |  |  |  |  |
| **Cor Total** | 0.4040 | 29 |  |  |  |  |  |

**Table S13** ANOVA of the regression model. (-OH scavenging capacity, methanol)

| Source | Sum of Squares | df | Mean Square | F-value | p-value | significant |  |
| --- | --- | --- | --- | --- | --- | --- | --- |
| **Model** | 3552.02 | 14 | 253.72 | 12.43 | < 0.0001 | ** |  |
| A-A | 519.31 | 1 | 519.31 | 25.44 | 0.0001 | ** |  |
| B-B | 56.61 | 1 | 56.61 | 2.77 | 0.1166 |  |  |
| C-C | 0.9761 | 1 | 0.9761 | 0.0478 | 0.8299 |  |  |
| D-D | 1531.84 | 1 | 1531.84 | 75.03 | < 0.0001 | ** |  |
| AB | 0.6972 | 1 | 0.6972 | 0.0342 | 0.8559 |  |  |
| AC | 41.73 | 1 | 41.73 | 2.04 | 0.1733 |  |  |
| AD | 912.64 | 1 | 912.64 | 44.70 | < 0.0001 | ** |  |
| BC | 1.97 | 1 | 1.97 | 0.0967 | 0.7601 |  |  |
| BD | 28.04 | 1 | 28.04 | 1.37 | 0.2595 |  |  |
| CD | 36.48 | 1 | 36.48 | 1.79 | 0.2012 |  |  |
| A² | 178.68 | 1 | 178.68 | 8.75 | 0.0098 | ** |  |
| B² | 3.81 | 1 | 3.81 | 0.1866 | 0.6719 |  |  |
| C² | 261.01 | 1 | 261.01 | 12.78 | 0.0028 | ** |  |
| D² | 2.02 | 1 | 2.02 | 0.0987 | 0.7577 |  |  |
| **Residual** | 306.25 | 15 | 20.42 |  |  |  |  |
| Lack of Fit | 83.52 | 10 | 8.35 | 0.1875 | 0.9880 |  |  |
| Pure Error | 222.73 | 5 | 44.55 |  |  |  |  |
| **Cor Total** | 3858.26 | 29 |  |  |  |  |  |

**Table S14** ANOVA of the regression model. (-OH scavenging capacity, ethanol)

| Source | Sum of Squares | df | Mean Square | F-value | p-value | significant |  |
| --- | --- | --- | --- | --- | --- | --- | --- |
| **Model** | 4348.74 | 14 | 310.62 | 11.48 | < 0.0001 | ** |  |
| A-A | 599.67 | 1 | 599.67 | 22.17 | 0.0003 | ** |  |
| B-B | 11.97 | 1 | 11.97 | 0.4424 | 0.5161 |  |  |
| C-C | 25.32 | 1 | 25.32 | 0.9360 | 0.3486 |  |  |
| D-D | 1606.68 | 1 | 1606.68 | 59.40 | < 0.0001 | ** |  |
| AB | 3.92 | 1 | 3.92 | 0.1448 | 0.7089 |  |  |
| AC | 72.62 | 1 | 72.62 | 2.68 | 0.1221 |  |  |
| AD | 677.71 | 1 | 677.71 | 25.05 | 0.0002 | ** |  |
| BC | 58.60 | 1 | 58.60 | 2.17 | 0.1617 |  |  |
| BD | 4.85 | 1 | 4.85 | 0.1794 | 0.6779 |  |  |
| CD | 0.0005 | 1 | 0.0005 | 0.0000 | 0.9967 |  |  |
| A² | 484.61 | 1 | 484.61 | 17.92 | 0.0007 | ** |  |
| B² | 139.05 | 1 | 139.05 | 5.14 | 0.0386 | * |  |
| C² | 940.36 | 1 | 940.36 | 34.76 | < 0.0001 | ** |  |
| D² | 70.11 | 1 | 70.11 | 2.59 | 0.1283 |  |  |
| **Residual** | 405.74 | 15 | 27.05 |  |  |  |  |
| Lack of Fit | 149.05 | 10 | 14.90 | 0.2903 | 0.9546 |  |  |
| Pure Error | 256.69 | 5 | 51.34 |  |  |  |  |
| **Cor Total** | 4754.49 | 29 |  |  |  |  |  |

**Figure S1** Response surface and contour of the effect of extraction time, the power of ultrasound, percentage of methanol in the water, and solid: liquid ratio on TSC.

**Figure S2** Response surface and contour of the effect of extraction time, the power of ultrasound, percentage of ethanol in the water, and solid: liquid ratio on TSC.

**Figure S3** Response surface and contour of the effect of extraction time, the power of ultrasound, percentage of methanol in the water, and solid: liquid ratio on TFC.
